# Personalised Medicine—Implementation to the Healthcare System in Europe (Focus Group Discussions)

**DOI:** 10.3390/jpm13030380

**Published:** 2023-02-21

**Authors:** Dorota Stefanicka-Wojtas, Donata Kurpas

**Affiliations:** 1 Clinical Trial’s Department, Wroclaw Medical University, 50-556 Wroclaw, Poland; 2 Family Medicine Department, Wroclaw Medical University, 51-141 Wroclaw, Poland

**Keywords:** personalized medicine, interregional cooperation, barriers, facilitators, healthcare systems

## Abstract

Background: Personalized medicine (PM) is an approach based on understanding the differences between patients with the same disease and represents a change from the “one size fits all” concept. According to this concept, appropriate therapies should be selected for specific groups of patients. PM makes it possible to predict whether a particular therapy will be effective for a particular patient. PM will still have to overcome many challenges and barriers before it can be successfully implemented in healthcare systems. However, it is essential to remember that PM is not a medical revolution but an evolution. Methods: Three focus groups were conducted, to achieve the purpose of this study, which was to identify the barriers and facilitators existing to the implementation of PM and to highlight existing practices in European countries. Focus group discussions covered the areas of barriers and facilitators to the implementation of personalized medicine. Results: This section describes the results of the focus groups that covered the areas of barriers and facilitators of personalized medicine implementation. Conclusions: Personalized medicine faces many challenges and barriers before it can be successfully implemented in health systems. The translation of PM to European countries, differences in regulations, high costs of new technologies, and reimbursement are the reasons for the delay in PM implementation.

## 1. Introduction

Personalized medicine (PM) is medical treatment that tailors prevention and treatment strategies for an individual patient. There is no universally accepted definition of personalized medicine.

However, in December 2015, EU Health Ministers published the following definition of PM in the Council Conclusions on Personalised Medicine for Patients: *a medical model that uses characterisation of individuals’s phenotype and genotype (e.g., molecular profiling, medical imaging, lifestyle data) for tailoring the right therapeutic strategy for the right person at the right time and/or to determine predisposition to a disease and/or to enable timely and targeted prevention* [1].

Indeed, as pointed out by Suwinski et al., growing attention is being paid to personalized medicine. This represents a fundamental shift from “one size fits all” methods of treating patients with diseases or predispositions towards new approaches, such as targeted therapies, which can achieve the best outcomes in treating patients’ diseases [2].

As Yeonhee Park stated, the era of personalized medicine is approaching, taking into account individual variability of genes and environment. In this era, it is crucial to consider the patients’ characteristics and demonstrate clinical benefits for patients [3].

As Jorge Alberto Bernstein Iriart noted, there is increasing investment in research in personalized and precision medicine. These investments are extremely important and require a large number of resources, as PM individualizes medical practice and puts people at the center, based on genetic testing, the identification of biomarkers, and the development of targeted drugs. However, the individualized or preclinical medicine movement is controversial and has led to significant disputes between its proponents and critics (mainly for financial reasons) [4].

The greatest cause for concern, according to Love-Koh et al., is that PM will change how some healthcare services are delivered and evaluated, even if the adoption of PM promises significant benefits. As he points out in his paper on the future of precision medicine, the lifespan of guidelines may become shorter, uncertainties in structure may increase, and new equity considerations will emerge. With the rapid increase in biomarker discovery and the advent of artificial intelligence (AI) technologies, improvements in methods and the evaluation of evidence will help achieve and maintain the goal of cost-effective healthcare. PM allows tailoring healthcare interventions to patient groups based on their disease susceptibility, diagnostic and/or prognostic information, or response to treatment [5].

One striking problem is that there has been a massive growth in data in the health sector in recent years. Some reports state that data generation in healthcare is increasing by 48% annually [6]. As Lopes-Júnior points out, the current challenge is to turn this expanded medical data collected in the health sector into clinical benefits for patients, by providing more predictive diagnoses, treatments, and personalized care to targeted individuals and populations [7].

Personalized medicine faces many challenges and barriers to being successfully implemented in healthcare systems. However, it is essential to remember that personalized medicine is not a medical revolution but an evolution. The concept of personalized medicine has been around for several decades, and the use of personalized approaches in medical treatment has steadily increased over time. The development of new technologies has accelerated the growth of personalized medicine in recent years. However, these advances are built on a foundation of scientific research and medical practice that goes back many years. In this sense, personalized medicine can be seen as an evolution of medical practice, rather than a sudden, revolutionary change. The development of personalized medicine is an ongoing process that builds on existing knowledge and technology and will continue to evolve with discoveries and the development of new technologies.

The benefits of personalized medicine are many, ranging from improving diagnostic accuracy to identifying the best treatment option for a patient based on their characteristics, to targeted therapy that increases the likelihood of successful treatments, reduces side effects, allows for better disease prevention, and most importantly, increases patient engagement, reduces healthcare costs, and also promotes research and innovation.

This paper discusses the barriers and facilitators to the implementation of personalized medicine interventions, and forms one of the outcomes of the Horizon 2020 project Regions4PerMed: “Interregional Coordination for a fast and deep Uptake of Personalised health”

## 2. Purpose of the Study

The purpose of the study was to identify the existing barriers and facilitators to the implementation of personalized medicine, to identify potential methods to address them, and to highlight the existing practices in European countries that work successfully to support the implementation of personalized medicine interventions.

## 3. Materials and Methods

### 3.1. Ethics Approval

This study was approved by the Bioethics Committee of the Medical University of Wroclaw under the number KB0450/2020.

### 3.2. Study Design

To achieve the aim of the study, three focus groups were conducted. The discussions in the focus groups were on the areas of barriers and facilitators to implementing personalized medicine.

### 3.3. Settings

The discussion of the online focus groups took place on the Zoom platform. The focus groups developed provided insights into people’s thinking and provided a deeper understanding of the phenomenon of personalized medicine. There were three categories of participants in the online focus group: observer, moderator, and respondent. During the focus group, questions were asked about individual understandings of personalized medicine; key facilitators and barriers to public use of PM; and how these related to respondents’ private opinions about the ease of adapting PM to citizens’ needs.

Specification of the questions:

Question 1: What (in your opinion) is personalized medicine?

Question 2: What are the most important facilitators and barriers to public use of personalized medicine? What are the barriers/facilitation related to?

Question 3: Can personalized medicine be easily adapted to the needs of the citizens? What could be helpful?

Details of the coding method design: participants’ country code, participant number (from 1 to 7), and focus group number (from 1 to 3).

### 3.4. Questionnaire Survey Development and Data Collection

#### Participants

The focus groups were conducted in three groups. The first focus group was conducted with representatives of Polish government institutions, financial institutions, representatives of patients’ rights, and patients’ foundations. There were 7 participants, apart from the observer and the moderator. The second group consisted of representatives of the European Commission, the Italian Ministry of Health, a scientist, and a general practitioner from Ukraine. The second focus group was attended by 4 people. The third group was held with representatives of the Saxon State Ministry of Science, Culture and Tourism and the Fondazione Regionale per la Ricerca Biomedica. Three persons participated in this meeting.

### 3.5. Variables

#### 3.5.1. Quantitative Variables

The focus groups provide information on the age, gender, and nationality of the respondents.

#### 3.5.2. Qualitative Variables

The focus groups generated empirical data on the individual experiences of respondents in relation to barriers and facilitators to the implementation of personalized medicine across Europe.

### 3.6. Data Sources

The presented study analyzed data from three online focus groups covering the barriers and facilitators to implementing personalized medicine. Focus groups were conducted on the following days 10 November 2022, 29 November 2022, and 12 December 2022. The focus groups lasted between 60 and 90 min. The data was collected locally on the server.

### 3.7. Study Size

The results of the focus groups are presented in this study.

## 4. Results

### 4.1. Descriptive Data—Focus Groups

This section describes the results of the focus groups that covered the areas of barriers and facilitators to the implementation of personalized medicine.

During the focus groups, a difference in the definition of personalized medicine became evident.

As one focus group participant pointed out, many meetings with scientists and medical professionals began with discussions about what should be called personalized medicine, precision medicine, individualized medicine, genomic medicine, etc. [BE.3.2].

The first respondent’s PM definition was individualized medicine based on genetic testing and tailored health interventions, in both preventive and restorative medicine, i.e., matching the best therapy for a person based on specific genetic predispositions [PL.1.1].

The next respondent used the definition published in the Council Conclusions in December 2015. This document contains a very broad definition of personalized medicine (the definition quoted in the introduction to this article) [BE.3.2].

According to another approach, personalized medicine exists at the moment when care takes into account personal risk factors and co-morbidities and decides what is best to prescribe to patients according to their situation, economic status, etc. There are already some international guidelines that have implemented personalized care in their approaches. For example, the American Diabetes Association has already included a personalized approach in the standards for diabetes care, when deciding what medications to prescribe and what type of prevention to offer patients, depending on their cardiovascular disease or risk factor, obesity, economic status, or kidney disease [UA.1.2].

Personalized medicine, for another respondent, means that doctors and nurses in any medical facility have information about patients, such as their age, previous diseases or conditions, information about allergies, etc., and that a diagnosis is made based on this information [IT.2.3].

As already mentioned, PM is the right therapy for the right patient at the right time, in the sense of the definition used by the European Council. Another very important aspect is data-driven personalized medicine [DE.1.3].

#### 4.1.1. Data Protection

The collection and compilation of genetic data raises several ethical issues, and there is also a risk of leaking genetic data as medical data, which has a relatively high risk. A major problem is also the compatibility of the data collected. There are a variety of local, small, medium, and large initiatives. The data collected has a different format and is defined differently. In addition, there is a lot of archival data that cannot be used due to a lack of appropriate permissions [PL.1.1].

It should be mentioned that genetic data can be used for positive purposes; if the entire genotype in addition to the blood group were recorded on the patient’s account, then on this basis, with this kind of information, it would be possible to shape the entire health policy in terms of their health needs. Scientific methods could be developed to find specific correlations between certain genes and health problems. Data collection also allows for scientific analysis [PL.1.1].

However, it was pointed out that for a worldwide database (e.g., for a bone marrow donor registry), global criteria must be met. The question was also raised as to whether it would not make sense to also examine and record the genotype when collecting umbilical cord blood from infants [PL.5.1].

In addition, it was noted that, in Poland, there is a database of basic medical data linked to paramedic and emergency department systems. According to this, a paramedic arriving at a patient’s home immediately knows what disease the patient has. Such databases have been checked with security services and can be expanded [PL.6.1].

#### 4.1.2. Data Analysis

It was also noted in the focus groups that personalized targeted therapies are often used in small groups, and small patient populations in very specific situations, which consequently creates many difficulties in obtaining reliable, validated data. There is no way to scientifically validate the evaluation of the efficacy of such therapy, which can continue for years under “unscientific” conditions for commercial purposes, often to the detriment of patients [PL.7.1].

#### 4.1.3. Government/Political Systems

One of the main problems with introducing personalized medicine to the global market is related to government at all levels (local/regional/national/European) and the government agencies responsible for the national regulatory framework and funding of national infrastructures with different responsibilities (health/education/research/innovation) [UA.1.2].

There is a need to provide measures for the population at the state and local level. It would be helpful if patients and GPs worked together with policymakers [UA.1.2].

Even though many political systems are aware of the benefits that personalized medicine can bring. For example, today people know how to store data and how to share data, it is not done because it takes time, because it requires skills that are not necessarily available, and because it requires not just a single change, but a change in the whole system [IT.2.3].

To show the decision-makers the way, it is essential to have good examples from other countries [DE.1.3].

#### 4.1.4. Healthcare Providers

The most important factor for introducing PM into a healthcare system is evidence. There is a need to demonstrate the evidence for PM and the legitimacy of its goals, and this is the most important issue for researchers, clinicians, and policymakers. There is also a clear need for more clinical trials [IT.4.2].

There is also a psychosocial issue; training health workers to use genetic data to share information about a possible stroke or heart failure, for example. Therefore, this “soft area” of research should be considered [PL.2.1].

#### 4.1.5. Financial Institutions

According to several respondents, cost is the most significant barrier to implementing PM. There is a fear in the minds of policymakers and funders that PM is a rich man’s medicine, and it is essential to fight against this, as it blocks the implementation of PM [BE 3.2], [FR.2.2], [UA.1.2].

However, funding therapies with guaranteed benefits or government-reimbursed medicines is problematic because the patient groups targeted by these therapies are often very small. Such narrowing of patient groups often leads to medicines being given orphan drug status, resulting in very high prices for these medicines and these therapies, which are difficult for the reimbursement system to bear [PL.7.1].

In contradistinction is the view that, in the PM context, we should not necessarily just talk about a broader approach that improves population health and makes better use of resources, not necessarily saving money, but one that makes better use of and improves health. A good example is pharmacogenomics. In this field, resources and money can be better used and lives can be saved by reducing the harmful effects of drugs [BE.3.2].

There is also a financial problem related to the funding and availability of genetic testing. Such tests are expensive and complicated. In addition, the availability of geneticists and the ability to use such data are limited [PL.7.1].

#### 4.1.6. Medical Doctors/Practitioners

One of the most important facilitators would be the implementation of an international directive and this approach in other protocols. This would enable more effective implementation of PM in medical practice [UA.1.2].

Training events need to be conducted to share knowledge with other countries. Such training should include the different tools, omics data, and possibilities [IT.4.2].

As highlighted by the interviewees, it is worth noting that policymakers are trying to make improvements to health care close to home or to territorial health care, whereas GPs focus on the person. The more data and the more technology there is, the better, but it is also crucial that health professionals have time to focus on one patient at a time [IT.2.3].

Some healthcare professionals resist change, are reluctant, and do not embrace certain aspects, at least at the moment. This may change with time and training [DE.1.3].

Another barrier to personalized medicine is the way doctors are trained today. They are very much focused on specific disease areas and there is a lack of awareness of other disciplines, which makes full adoption of personalized medicine require a comprehensive medical approach. Thus, the training of doctors is an important factor and, if adopted, could also be an important facilitator [DE.1.3].

#### 4.1.7. Healthcare Systems

The transformation of health organizations is also important. It is not only the training of nurses and doctors that is needed, but also the training of other professional groups that can be integrated into a hospital, to additionally support the implementation of personalized medicine [IT.2.3].

One suggestion is the introduction of so-called case managers or patient companions, people who support patients in the system, especially if they have a very threatening disease. With the depth added by personalization in healthcare, there is a need for counselling for patients and the question arises whether this must be done by a doctor or whether someone with a good medical training background might be able to help or provide guidance in the system [DE.1.3].

#### 4.1.8. Patients

One of the many noticeable barriers to implementing PM is the personal barrier [UA.1.2].

Patient involvement in research is important. Projects are and should be more patient-centered [IT.4.2].

It is important to push for patient involvement in every step of health care and research, even if the budget is not always available [IT.4.2].

The population should become aware of PM, and of the best practices that could create this. Citizens should be better educated about what is currently possible and what PM will bring to the system [DE.1.3].

#### 4.1.9. Technological Developments

It is very important to improve digitalization. There is also a lack of health technology assessments that properly show the benefits [DE.1.3].

## 5. Discussion

Precision medicine is an approach to healthcare that uses personal information, including genetic, environmental, and lifestyle data, to improve disease prevention, diagnosis, and treatment. In addition to the positive potential of precision medicine, there are concerns about data sharing, patient privacy, and equal access to treatment. The enormous potential and rapid pace of innovation, combined with the expected risks and unintended consequences, make precision medicine an ideal technological area for agile governance. Using the latest technologies to improve treatments, store patient data, and track outcomes is essential for a country to achieve its healthcare goals under changing environmental, economic, and social conditions [8].

### 5.1. Government/Political Systems

Translating PM to the governance of health care is an important problem, a challenge for policymakers, and a political issue. It requires regulatory efforts, such as frameworks to protect patients and citizens from discrimination based on their genetic profile. It also requires regulation and the creation of incentives for providers and funders to develop products for the medical market [9].

One problem for Europe is the cross-border sharing of patients, biological material, and technical resources. Not all legal issues for optimal cooperation have been clarified, such as the need to transport patient data and biological material across borders [10].

PM is included in national regulations, plans, or EU-MS strategies, depending on the country, in line with the recommendations of EC. Italy has been a pioneer in implementing PM in healthcare, through specific national plans for genomics in public health genomics and omics science in public health; the United Kingdom through genomics, personalized prevention, and citizen engagement; and Estonia, through innovative strategies and biobanking [11].

### 5.2. Data Sharing

A critical component of personalized medicine is collecting, storing, and using genomic and clinical data from patients and healthy patients. To maximize the value of this data, it is important to create a culture in the scientific, medical, and patient communities that promotes the proper sharing of genomic and clinical information [12].

In addition, when it comes to data comparability, an important question for regulators is how to regulate data sharing among different stakeholders, particularly patients, physician practices, hospital providers, pharmaceutical and clinical researchers, and health insurers. Data sharing in PM is not only relevant for health systems in one country, but also for data exchange between different countries. This issue is a challenge for high-, low-, and middle-income economies [9].

Research has shown that there is a great need for mutual recognition for medical digital solutions published in other EU Member States [13].

### 5.3. Education

The training of doctors is generally still very old-fashioned and focuses on reactive treatment. In order to keep up with change, the various actors within the health system need to be trained in a completely different way. Central to this is the ability to work as part of a multidisciplinary team that includes doctors, nurses, medical imaging engineers, and others who collect information from patients [14]. After all, health professionals and citizens will determine the future of PM through their involvement, participation, and interaction in policy making. Despite increasing educational strategies, certain aspects still need to be improved, in terms of accessibility, target groups, or the tools and methods used [11].

For this reason, education and discussions on the concept of personal health are necessary for a large forum of politicians and citizens. We note that there is a lack of education of physiotherapists, as well as awareness and motivation among the population [15].

### 5.4. Healthcare System

According to the assessment proposed in the literature, the integration of PM into national health systems should be based on six key themes: the healthcare system, governance, access, awareness, implementation, and data. The governance dimension should include both a national strategy and comprehensive dimensions of legislation; policies; and ethical, social, and legal frameworks that address personalized medicine and genetic data sharing. On a large scale, research initiatives (national research center) should also be launched and there should be legislation for consumer testing or a code of conduct for consumers. Study groups involving all stakeholders interested in the introduction of PM should be conducted. It should also be noted that the introduction of PM into existing healthcare systems requires matching emerging new services and practices that are aligned with both national regulations and national funding systems [9].

### 5.5. Financing

Personalized medicine is a new discipline that is just entering the market. Therefore, knowledge about the economic importance of PM is unfortunately not yet well developed. As Kalouguina et al. note, the literature points precisely to the insufficient quantity of real-world data, i.e., the cost-effectiveness of a personalized medicine or treatment after it has been implemented in clinical practice. Of the 26 studies reviewed by the authors that mentioned economic relevance, more than 60% pointed to a lack of studies evaluating the applicability of PM. Some of the studies mentioned the problem of funding PM, but some of the articles emphasized that this is precisely because of the lack of evidence. PM needs to overcome this lack of evidence to reach its optimal potential. In the absence of clear data on the cost-effectiveness of PM, funders do not have the slightest motivation to reimburse it, as it has not been proven that PM is profitable. As funders admit, their skepticism also stems from uncertainty about the clinical benefits of drugs and PM technologies [16].

This is because the evidence currently available is scarce and quite heterogeneous in terms of its quality and the application of common rules for documentation. Therefore, it is considered insufficient for funders to offer coverage. Therefore, there is no standard of evidence, and experts navigate cost–utility and cost–effectiveness analyses with different outcomes, such as cost per quality-adjusted life-years (QALYs), cost per life-years gained, or incremental cost-effectiveness ratios [16].

PM is considered a tailor-made prevention and treatment strategy for individuals or groups, so that patients receive specific therapies that are most appropriate for them and no money is wasted on trial-and-error treatments [17].

## 6. Limitations of the Study

The analytical aim of the study presented in this paper may have some limitations.

A limitation of focus groups is that the moderators may tend to generalize or categorize the individual feedback into group sentiment. To prevent this, an observer participated in the focus groups, in addition to the moderator. In addition, people with a strong influence, such as the moderator and vocal group members, can influence the conversation and make it seem one-dimensional or suppress feedback from less vocal participants. To avoid this factor, the group moderator tried to moderate the conversation, so that each participant could express their opinion. To avoid this issue, three focus groups were conducted, involving government policy representatives, health care providers, insurance policy makers, patient foundation representatives, and general practitioners.

Another limitation that can be considered is the content of the questions asked in the focus groups. However, the content of the questions was approved by a supervisor, who is a professor experienced in qualitative and quantitative research.

## 7. Conclusions

Personalized medicine has received significant attention in the last decade, as technologies for understanding biological differences between individuals have advanced significantly [18].

As highlighted in the introduction of this article, personalized medicine still has many challenges and barriers to overcome before it can be successfully implemented in healthcare systems.

Despite its measurable benefits, the complicated process of translating PM to EU member states and European health systems is delaying its widespread adoption [19].

There are numerous potential benefits of personalized medicine, including minimizing the risk of drug toxicity, increasing the benefits of the medicines used, contributing to the balance of the healthcare system, and facilitating drug discovery and development programs [18].

Unfortunately, there are also several barriers to smooth PM implementation, such as cost [13] (the high cost of new biotechnologies can exacerbate health inequalities and become a problem for the sustainability of health services, especially in low- and middle-income countries [4]), complexity, requirements for high-quality evidence, and the need for further training, which have so far limited the clinical implementation of pharmacogenomic testing [18].

In addition, data protection regulations and differences in regulation across European countries are problematic. Issues that need clarification were also discussed, such as the regulatory requirements for evidence for pharmacogenomic testing and the need for multiple pathways and pharmacogenomic marker development [18].

## Data Availability

Restrictions apply to the availability of these data. Data was obtained from third party and are available from the authors only as an anonymised version.
